# Lidocaine patch (5%) is no more potent than placebo in treating chronic back pain when tested in a randomised double blind placebo controlled brain imaging study

**DOI:** 10.1186/1744-8069-8-29

**Published:** 2012-04-24

**Authors:** Javeria A Hashmi, Marwan N Baliki, Lejian Huang, Elle L Parks, Mona L Chanda, Thomas Schnitzer, A Vania Apkarian

**Affiliations:** 1 Department of Physiology; 2 Department of Rheumatology; 3 Department of Anesthesia, Surgery, Northwestern University, Feinberg School of Medicine, Chicago, Illinois 60611, USA

**Keywords:** Chronic pain, fmri, Clinical trial, Placebo, Lidocaine, Topical analgesic

## Abstract

**Background:**

The 5% Lidocaine patch is used for treating chronic neuropathic pain conditions such as chronic back pain (CBP), diabetic neuropathy and complex regional pain syndrome, but is effective in a variable proportion of patients. Our lab has reported that this treatment reduces CBP intensity and associated brain activations when tested in an open labelled preliminary study. Notably, effectiveness of the 5% Lidocaine patch has not been tested against placebo for treating CBP. In this study, effectiveness of the 5% Lidocaine patch was compared with placebo in 30 CBP patients in a randomised double-blind study where 15 patients received 5% Lidocaine patches and the remaining patients received placebo patches. Functional MRI was used to identify brain activity for fluctuations of spontaneous pain, at baseline and at two time points after start of treatment (6 hours and 2 weeks).

**Results:**

There was no significant difference between the treatment groups in either pain intensity, sensory and affective qualities of pain or in pain related brain activation at any time point. However, 50% patients in both the Lidocaine and placebo arms reported a greater than 50% decrease in pain suggesting a marked placebo effect. When tested against an untreated CBP group at similar time points, the patch treated subjects showed significantly greater decrease in pain compared to the untreated group (n = 15).

**Conclusions:**

These findings suggest that although the 5% Lidocaine is not better than placebo in its effectiveness for treating pain, the patch itself induces a potent placebo effect in a significant proportion of CBP patients.

## Background

Multiple lines of evidence suggest that aberrant activity in sodium channels contributes to chronic pain conditions that involve neuropathy. Blocking sodium channels such as with systemic Lidocaine reduces evoked intensity of acute pain [1-5] and also relieves pain in some patient populations such as in chronic post herpetic neuralgia [2,6,7]. The main mechanism through which Lidocaine is said to act is by inhibition of ectopic discharge in sensitized and hyperactive cutaneous nociceptors. In addition, a central analgesic effect of Lidocaine has also been suggested [5,8].

The most advocated mode of Lidocaine administration is with 5% Lidocaine adhesive patches that are applied to the affected area and act by local absorption [9,10]. The systemic absorption is minimal; hence the chances of adverse side effects are low. However, the effectiveness of patches medicated with Lidocaine (5%) in reducing pain is less clearly understood. Some recent studies reported that 5% Lidocaine patches either have variable effects or no effects in acute pain models of pain in healthy subjects suggesting a partial and inconsistent block of nociceptors [11-13]. These findings raised important questions regarding the mechanisms and potential efficacy of Lidocaine patches in reducing pain and led to the speculation that Lidocaine patches may be more effective in reducing pathological pain related with an abnormally increased expression of sodium channels [14]. This assumption is based on the fact that chronic pain patients with neuropathic pain conditions such as low back pain, painful diabetic neuropathy and complex regional pain syndrome benefit from treatment with topical Lidocaine, but even in these conditions, the effects of Lidocaine on pain are variable between subjects and can range from 29% to 80% of the studied cases [2,11,14-18]. Whether Lidocaine patches are effective in treating chronic back pain is particularly unclear since the purported effectiveness of the treatment is derived from open labelled clinical trials [18-21]. fMRI studies have also shown central analgesic effects of 5% Lidocaine, but again these studies were not controlled for a placebo effect [18,20,22-25].

It is not known whether the 5% Lidocaine patch has a true pharmacological effect on chronic back pain or if it is a potent placebo. Here we aimed to study how the 5% Lidocaine patch compares with a placebo patch in reducing pain of CBP and to investigate brain activity that can differentiate between the treatments. We hypothesised that pain and related brain activity will diminish more in participants in the active treatment arm. In a randomised double-blind, placebo controlled clinical trial, 30 CBP patients received drug or placebo treatment and underwent brain imaging to identify activity for fluctuations of spontaneous pain, at baseline and at two time points (6 hours and 2 weeks) after start of treatment. Moreover, we investigated inter-individual differences in pain responses to test whether a subset of patients is more responsive to Lidocaine treatment than placebo treatment.

## Results

### Effects of Lidocaine vs. placebo on CBP pain

The chronic back pain intensity (peak pain in spontaneous pain ratings on numerical 0-100 scale and visual analog 0-10 scale values) and back pain properties (sensory, 0-33 scale, and affective, 0-12 scale, pain qualities from MPQ) were compared between the two treatment groups at baseline, 6 hours, and 2 weeks post treatment using a repeated-measures two-way (drug and placebo treatment arms by sessions) repeated-measures analysis of variance (2-RM-ANOVA). The peak rating of spontaneous pain and the VAS scores were strongly correlated with each other (R = 0.53, p ≪ 0.002, n = 30), and since the spontaneous pain was collected during fMRI acquisition, we designated its peak pain rating as the pain intensity criterion for brain activity. Also note that at baseline, there was no significant difference (p ≫ 0.05) between the two groups in depression scores (BDI), anxiety scores (BAI) or neuropathic pain scores (NPS).

**Figure 1:**
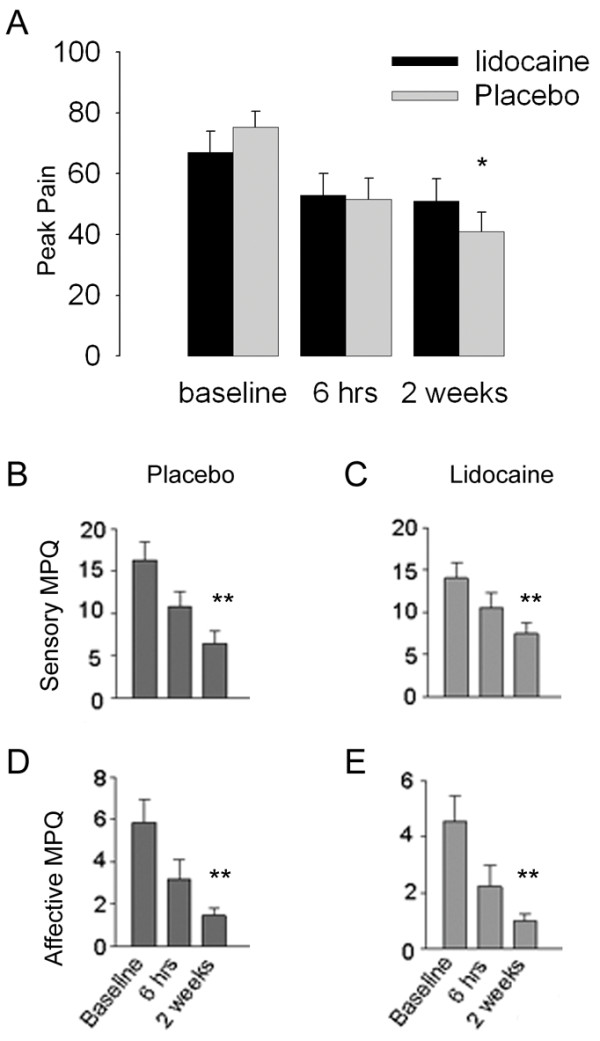
**Pain did not differ between chronic back pain (CBP) patients treated with 5% lidocaine patches or with patches containing no active drug (placebo). A.** Variation of CBP pain with treatment type and treatment duration. Treatment duration, but not type, significantly decreased CBP pain. **B-E.** Effect of treatment type and duration on sensory (range 0-33) and affective scores (range 0-12) obtained on the McGill pain Questionnaire (MPQ). Sensory and affective scores decreased with treatment duration for both types of treatment. Error bars represent SEMs. * p ≪ 0.05, ** p ≪ 0.01 differences from baseline.

There was a significant decrease in back pain magnitude with treatment duration (F _2,89_ = 7.8, p ≪ 0.001) but no treatment type effect (F_1,89_ = 0.72, p = 0.4), and no significant interaction between type and duration of treatment (F _2,89_ = 0.03, p = 0.99). At baseline, the lidocaine treated group did not show a significant difference in back pain magnitude from the placebo group (Figure 1A). Similarly, there was no treatment type effects at the 6 hour (F _1,29_ = 0.18, p = 0.89) and 2 week period (F _1,29_ = 1.06, p = 0.31).

For both Lidocaine and placebo treated groups, there was a decrease in the sensory and affective MPQ scores for treatment duration (Sensory: F_2,84_ =11.6, p ≪ 0.0001; affective F_2,84_ = 22.66, p = 0.0001), but there was no treatment type effect at 6 hours (sensory p ≫ 0.5; affective p ≫ 0.3) or at 2 weeks (sensory p ≫ 0.1; affective p ≫ 0.4) (Figure 1B-E). Overall, the effects of Lidocaine patch on CBP pain could not be distinguished from that of placebo. Yet, we observe decreased pain of CBP with continued treatment for both treatment groups. Note that there were no harmful or unintended effects reported by subjects in either group.

### Effects of Lidocaine vs. placebo on CBP pain related brain activity

**Figure 2:**
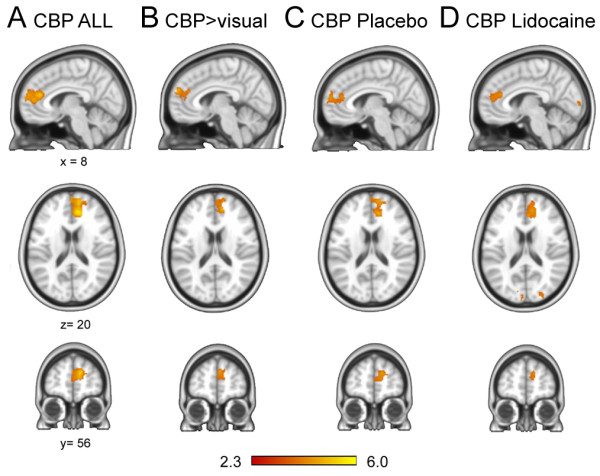
**Different groupings for brain activity for spontaneous fluctuations of pain of CBP calculated for brain scans collected at baseline.** Coordinates x = 8, y = 56, z = 20 for A**-D** (top row are sagittal, middle horizontal, and bottom coronal slices; middle and bottom rows: left side is left hemisphere). **A.** Whole-group average brain activity for rating spontaneous pain of CBP patients (n = 30 subjects). Brain activity was limited to medial prefrontal cortex (BA 9) and the genual anterior cingulate cortex (BA 32). **B.** Contrast between activity for rating spontaneous pain of CBP and rating length of a bar varying in time (control for visual, motor, and task demands; paired t-statistic n = 30 subjects) identifies the same brain activity as in A. **C and D.** Brain activity was similar between placebo (C) and lidocaine (D) treated groups for spontaneous pain of CBP at baseline (n = 15 subjects per group), and closely matched whole-group activity shown in activity and contrast maps were generated using random-effects statistics with z score ≫ 2.3 and cluster threshold p ≪ 0.01, corrected for multiple comparisons.

Average group activity map was generated for 30 CBP patients to determine brain regions reflecting spontaneous back pain. The spontaneous pain ratings correlated significantly with BOLD response in the medial prefrontal cortex, extending from the medial frontal pole to the genual anterior cingulate cortex (Figure 2A, Table 1). To correct for task related brain activity confounds we subtracted the visual rating activity maps from the pain rating activity maps (whole-brain paired t-test). The resultant map showed essentially the same pattern as the uncorrected map (Figure 2B) (opposite contrast was null). Furthermore, contrasting between the Lidocaine and the placebo treated groups (unpaired t-tests, n = 15 subjects per group) at baseline, at 6 hours, and at 2 weeks of treatment showed no significant brain activity. The mean activation maps for Lidocaine treated and placebo treated groups (n = 15 subjects per group) at baseline again showed spontaneous pain related statistically significant activation in the medial prefrontal and the genual anterior cingulate cortices (Figure 2C &2D). However, there was no significant mean activation at 6 hrs or at 2 weeks of treatment in either the Lidocaine treated group, or the placebo treated group. Overall, we observe a consistent brain activity at baseline for spontaneous pain of CBP. However, as back pain magnitude decreases with treatment the related brain activity also decreases in both groups.

**Table 1:** Patient clinical characteristics

	**Age**	**Duration**	**Sex**	**BDI**	**BAI**	**NPS**	**Sensory MPQ**	**Affective MPQ**	**MQS**	**Pain**
Mean	51.36	14.2	14 F	6.6	12.3	55.0	16.5	5.5	4.8	71.6
SD	9.08	12	16 M	4.2	8.8	15.5	7	3.6	6.1	24.1
Scale/Range	years	years		20	0-63	0-100	0-33	0-12	0-21	0-100

BDI, Beck Depression Inventory; BAI, Beck Anxiety Inventory; NPS, neuropathic pain scale; MPQ, McGill Pain Questionnaire; MQS, Medication Quantification Scale.

### Inter individual differences in patch induced analgesia

**Figure 3:**
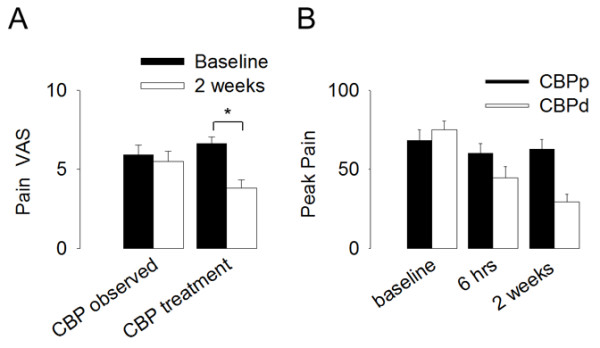
**Pain for treated and observed groups, and pain when treated group was subdivided based on pain of CBP decreasing (CBPd) or persisting (CBPp) after 2 weeks. A.** Pain at baseline and after 2-weeks (visual analog score, VAS, 0-10 score) in CBP patients who received no interventions or treatment instructions, CBP _*observed*_ (n = 15), in contrast to the patients who participated in the clinical trial for an ineffective treatment, CBP _*treatment*_ (n = 30). The two groups started at a similar intensity of back pain but only the CBP _*treatment*_ group showed decrease in back pain after two weeks. Error bars represent SEMs. * p ≪ 0.05. **B.** Back pain intensity, in CBPd and CBPp groups, as a function of treatment duration. A median split shows that on average the group that showed absolute pain change more than the median had significantly lower pain at the 2 week time point.

As a next step, we tested the hypothesis that a subset of susceptible CBP patients benefits more from Lidocaine treatment than the placebo treatment. For this analysis, first all subjects were separated objectively into two groups based on a median split. The median of the absolute change in pain in all subjects was 29.4. Thus, all subjects that showed more than median change in pain were designated to the CBP decreasing and those less than median pain change were assigned to the CBP persisting groups. Next, we investigated whether the percent change in pain differed between Lidocaine and placebo treated subjects within a subset of patients. There were eight placebo and 7 Lidocaine treated CBP subjects that had reported a more than median (median = 29.4) decrease in pain after 2 weeks of treatment and the mean percent change = 61.4%, SEM = 2.04. In the remaining subjects (below the median value) there were 7 placebo and 8 Lidocaine treated subjects and the mean percent change in this group was 3.9%, SEM = 0.13 (Figure 3B). Note that with this grouping, there was no difference in pain between the groups at the 6 hour time point (F_1,29_ = 2.7, p = 0.11).

Next, we compared the change in pain between the Lidocaine and placebo treated groups within the subset of subjects that showed a more than median decrease in pain i.e. the CBP decreasing group. The mean percent change in pain in the Lidocaine treated group (54.7%, SEM = 9.81) was not significantly different (t = 0.25, p = 0.8) from the placebo treated group (59.7% SEM = 10.316). Moreover, pain related brain activity was not significantly different between the placebo and Lidocaine treated subjects within the CBPd subset with a less stringent fixed-effects contrast. Using the same technique, contrasting brain activity between the Lidocaine and placebo treated subjects within the CBPp group showed no significant difference between the two groups.

### Is the patch a potent placebo?

The effects of 5% Lidocaine patch were indistinguishable from the placebo patch, but one remaining question was the marked reduction in pain observed in both Lidocaine and placebo treated groups. A greater than 50% reduction in clinical pain in a large proportion of subjects represents a marked effect and this analgesia could have been caused by a number of factors associated with the experiment. For instance, the clinical trial setting, expectation of pain relief from a treatment and the twice daily application of the patch for two weeks may have acted as a potent placebo. Alternatively, the reduction in pain in the CBPd group may have been due to other disease related factors such as due to natural fluctuations in pain intensity. Therefore, we compared back pain between the observational group (CBP _*observed*_, n =15) and the treatment group (CBP _*treatment*_, n = 30), at baseline and after two weeks. Within this time period, VAS rating for back pain significantly decreased for the CBP _*treatment*_ but not for the CBP _*observed*_ group (2-RM-ANOVA for the two groups and visits F _2,43_ = 8.33, p = 0.03; post-hoc comparisons show 1. no difference between groups at baseline, p ≫ 0.1, 2. no difference between baseline and 2-weeks for CBP _*observed*_ group p ≫ 0.6, 3. a significant decrease in VAS pain for CBP _*treatment*_ group between baseline, 6.6 ± 0.07, and 2-weeks, 3.8 ± 0.09, t_58_ = 4.3, p ≪ 0.001) (Figure 3A). Note that between the two groups there was no difference in 1) back pain duration (CBP _*treatment*_ 14.2 ± 0.39 years, in contrast to CBP _*observed*_ 14.5 ± 0.5, t-test p ≫ 0.9), 2) a borderline difference in age (t-test, p = 0.06), 3) no difference in gender (Mann Whitney rank sum test, p = 0.4), and 4) no difference in depression (t-test, p ≫ 0.4), attesting to the close match between the treatment and observed CBP groups. Therefore, we conclude that the presence of the potential analgesic treatment within the clinical trial setting was critical for the decrease in back pain intensity observed after two weeks in the CBP _*treatment*_ group.

## Discussion

Here we demonstrate that 5% Lidocaine patch reduces the magnitude of CBP through a mechanism that cannot be distinguished from the effects of the placebo patch. However, pain intensity was reduced in a significant proportion of subjects in the 5% Lidocaine and placebo treated groups. These findings indicate that the therapeutic effectiveness of 5% Lidocaine observed in other back pain studies was due to the potent placebo properties of the patch itself and not due to a pharmacological action of the drug.

Placebo controlled clinical trials have shown that systemic or topical Lidocaine reduces severity of chronic post-herpetic neuropathy, neuropathic pain, and for pain associated with inflammatory bowel disease [4,26,27]. This is the first placebo controlled clinical trial for 5% Lidocaine in chronic back pain and our findings indicate that the analgesic effects of 5% Lidocaine patch on CBP could not be distinguished from the placebo patch. In addition, there was a generalized decrease in sensory and affective pain qualities after treatment, but even in these measurements, the 5% Lidocaine treated group was not significantly different from the placebo group. In other clinical conditions such as painful diabetic neuropathy and complex regional pain syndrome, the drug showed greater benefit than placebo, but the effectiveness was variable ranging from 29% to 80% of studied cases [11,16-19].

The mode of action of topical Lidocaine is not clear and clearly shows inter individual variability in responsiveness between patients with neuropathic pain syndromes and also in evoked pain responses in healthy subject after treatment. In one study, several patients with complete loss of electric nerve function and marked subepidermal nerve-fiber plexus denervation in the peripheral limb showed a response to the Lidocaine patch [21]. An important implication of this study was that electric nerve function is not an essential for the mechanisms of 5% Lidocaine therapeutic action. Even in healthy subjects, 5% Lidocaine was not more effective than placebo in treating experimental pain and innocuous sensation including heat evoked pain, mechanical pain and capsaicin induced pain [11-13]. These negative findings led to the speculation that the 5% Lidocaine is too low a dose to effectively block healthy nociceptors, but may block pathological activity associated with upregulated sodium channels that result in neuropathic pain [11,14]. The Lidocaine patch has been suggested to affect neuropathic pain by a local non selective stabilization of sodium channels on cutaneous afferents at or near the site of application [1,9]. The findings of the present study corroborated by other studies raise some questions in this regard and show that Lidocaine was not more effective than placebo in treating chronic back pain that does have a significant contribution from neuropathic sources.

The 5% Lidocaine patch is an off label treatment for chronic back pain. This treatment has been increasingly advocated due to its purported effectiveness and is recommended over other treatments due to fewer side effects [19,28,29]. The confidence in the efficacy of the 5% Lidocaine patch especially for treating CBP is based mainly on open labelled trials and the role of placebo analgesia in mediating the actions of the 5% Lidocaine patch had not been tested before. Our findings suggest that the 5% Lidocaine patch acts as potent placebo and has no detectable pharmacological effect in either pain report or in brain activity. The fact that a nearly equal number of subjects in the Lidocaine and placebo arm reported a marked decrease in pain indicates that the effects of just the patch itself irrespective of the presence of drug can produce analgesia through endogenous pain regulatory mechanisms associated with placebos [30-32]. A putative placebo mechanism in reducing pain is reflected by the fact that only the patch treated group as a whole showed a significant reduction in pain intensity when compared to a group of CBP patients that were not given any treatment. This observation explains the positive findings we had reported in a previous report where in an open labelled trial, the 5% Lidocaine patch was effective in reducing pain intensity on average accompanied with related changes in pain related brain activation patterns after treatment [20]. However, in the present and in the preliminary study, we observed a marked reduction in clinical pain after treatment that suggests that the patch induces a potent placebo analgesia.

However, the present study also demonstrates that not all subjects responded with analgesia to the patch, and the percent change in pain was negligible in half of the subjects. Thus, the placebo effect induced by the patches is subject to a prominent inter-individual variability and this extent of variability has not been observed in previous placebo studies [31,33-35]. This could be because most placebo studies have studied healthy subjects and placebo responses in clinical populations may be affected by disease chronicity. Another prominent factor is that unlike most placebo studies where a group of subjects is conditioned to believe in the benefits of the treatment [36,37], here all patch treated subjects were given similar open ended instructions that the treatment may or may not reduce their pain. Thus the psychobiological mechanisms that lead to reduction or no reduction in pain would be reflective of each individuals own expectations, belief in the treatment and anxiety about the treatments potential benefits.

One limitation of this study is that the number of subjects is lower than what would be required for a clinical trial. However, corroborated by other studies, these findings indicate that a large sample size would result in a similar outcome. A calculation for the required sample size to achieve a clinically relevant change in pain of 20 % combined with the present findings required 536 subjects to achieve a desired power of 0.85 (large effect size). Such a large number of chronic back pain subjects would be extremely difficult if not unachievable to recruit for an fMRI study. Nevertheless, additional studies are needed that test the effects of the 5% Lidocaine patch against a placebo to arrive at a solid conclusion regarding the efficacy of this treatment in chronic back pain.

Recently, the 5% Lidocaine patch has emerged as first line therapy and since side effects are lower than oral or systemic doses, its use has become popular especially in geriatric populations. Our findings raise some important considerations since even though the 5% Lidocaine had no direct effect; the patch itself induces analgesia that is two to three folds higher than the accepted clinical level of 20% , but a discussion about the ethics of using placebos that produce strong analgesic effects is beyond the scope of the objectives of this study. This study brings to bear “the elephant in the room” issue relating to the ever present placebo effect in analgesic trials. This study also raises the need for more consideration into whether the clinical use of the Lidocaine patch in CBP is warranted. Overall, based on these findings, we conclude that the 5% Lidocaine patch has no drug mediated action on intensity of CBP; however, it does reduce pain intensity in more than 50% of subjects that is most likely due to a placebo effect. Our findings suggest that the patch is a potent agent for inducing placebo analgesia.

## Methods

### Subjects

**Figure 4:**
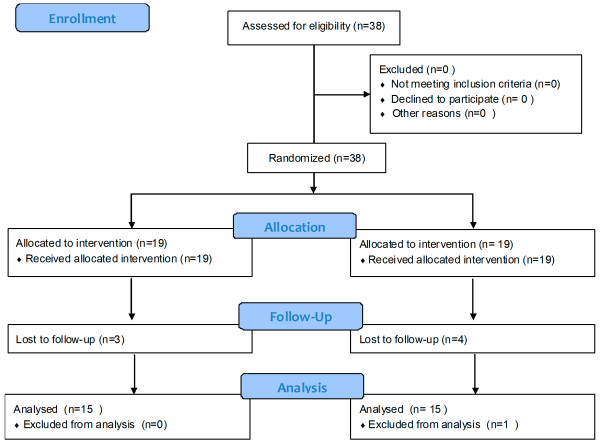
Consort 2012 flowchart.

A sample size of 30 subjects was predetermined based on a a review of literature [4,38]. A total of 38 patients were recruited for the brain imaging and treatment study. As shown in the consort flow chart shown in Figure 4, data from 7 subjects was not analyzed due to failure to attend the repeat sessions for non specific reasons and data from 1 subject was excluded from analysis due to technical faults. Thus, data from a total of 30 subjects (16 males, 14 females, age 51.36 ± 0.30 years, mean ± SEM) was included in the brain imaging analysis. An additional 15 patients (10 males, 5 females, age 46.3 ± 0.38 years) were recruited and their pain was measured at intervals similar to the patch treated groups. Brain imaging data was not collected in this group.

All subjects were right-handed and gave informed consent to procedures approved by the Northwestern University Institutional Review Board. Participants were compensated financially for their time. All patients, recruited by newspaper ads in Chicago area, were diagnosed with CBP by a clinician and had to fulfill a specific list of inclusion/exclusion criteria. Patients were included if they had CBP for ≫1 year, and a pain score ≫ 4/10 VAS at the baseline visit. Subjects were excluded if they suffered from co-morbidities, major psychiatric conditions or other medical conditions (Table 2). Patients who entered the brain imaging and treatment group were given specific instructions regarding the potential pain relief by the patch and were also told that they had a 50% chance of receiving either patches containing drug or placebo. During the 2 weeks of treatment period, they could take up to 2 regular strength acetaminophen tablets (325 mg) per day, if needed. All patients were asked to refrain from taking analgesic medications for 72 hours prior to the imaging session. Patients who entered the observational group were not given any instructions, were not administered any treatments, and were instructed that they can manage their pain by any means they deemed necessary.

**Table 2:** Coordinates of brain regions activated in relation to spontaneous ratings of CBP

	***Brain Region***	***Z-VALUE***	***co-ordinatesx y z***			***P - values***
CBP baseline activity pain task	r MPFC/gACC	5.29	12	56	22	0.001
	(BA 9, 32)					
CBP baseline activity	r MPFC/gACC	4.61	12	42	24	0.004
pain task>visual control	(BA 9, 32)					
Lidocaine: CBP baseline activity	r MPFC/gACC	3.66	16	36	20	0.004
pain task	(BA 9, 32)	3.25	6	42	18	0.040
Placebo: CBP baseline activity	r MPFC/gACC	3.95	12	38	18	0.0003
pain task	(BA 9, 32)					

### Subject groups and experimental sessions for brain imaging with treatment

Efficacy for pain relief by 5% Lidocaine patch was tested in a randomised, double blind, placebo controlled longitudinal study. Of the patients recruited to this part of the study, data from 15 CBP patients that received the patches containing 5% Lidocaine and 15 age and sex matched CBP patients that received a patch containing the vehicle and no Lidocaine (placebo arm) were included in the analysis. Participants were randomly selected to receive drug or placebo. The Northwestern University Clinical Unit personnel generated the random allocation sequence using a random number generator, and held its key to the end of the study. All patients and experimenters (while delivering treatments, scanning and analyzing data) were blinded to type of treatment. The first application of the unlabeled patch was carried out by a clinician (blinded to type of treatment) who also explained the proper use of the patch. The patient was supplied with a measured number of unlabeled patches (identical between drug and placebo arms) and specific instructions were given to self administer the patch twice daily at 12 hour intervals for a period of two weeks. There was no difference in the appearance of the patches that contained Lidocaine or no drug.

### Experimental tasks and fMRI data acquisition

Each volunteer in this group participated in three experimental sessions. The first session was conducted immediately before start of treatment (baseline), the second session was performed 6 hours after application of the first set of patches and the third session was after 2 weeks of using the patches. At baseline, the patients filled out questionnaires related to their pain that included the McGill pain questionnaire (MPQ), neuropathic pain scale (NPS), Beck depression inventory (BDI) and Beck anxiety inventory (BAI). Before scanning, participants were trained on a finger-span device that was later used for acquiring continuous ratings of the fluctuations of spontaneous pain of CBP, on a numerical scale ranging from 0-100 during functional scans. This device was composed of a potentiometer the voltage of which was digitized and time-stamped in reference to fMRI image acquisition and connected to a computer providing visual feedback of the ratings [39,40]. In addition to the pain-rating task, subjects were trained to perform a visual rating task [39] during which subjects rated the changes in the length of a bar on the 0-100 numerical rating scale projected on a screen. The length of the bar varied over time to match the pain ratings obtained from the subject in the preceding scan. Thus this task serves as a control for task-related activations such as visual inputs, motor performance, magnitude estimation, attention, and anticipation.

After training, the subjects were placed in the scanner, T1-weighted structural images and fMRI data were collected while subjects performed pain or visual rating tasks In addition to the pain rating scan, a visual rating task scan was acquired in which the subject rated the length of the bar as it varied over time in conjunction with the subjects own pain ratings obtained in one of the preceding spontaneous pain rating scans.

fMRI data were acquired with a 3-T Siemens Trio whole body scanner with echo-planar imaging (EPI) capability using the standard radio-frequency head coil. Multislice T2*-weighted echoplanar images were obtained with the following parameters: repetition time (TR) = 2.5 s; echo time (TE) = 30 ms; flip angle = 90°, slice thickness = 3 mm, in-plane resolution = 64 × 64. The 36 slices covered the whole brain from the cerebellum to the vertex. A total of 244 volumes were acquired per condition in all participants and the first 4 volumes were discarded during the preprocessing step. A T1-weighted anatomical MRI image was also acquired for each subject using the following parameters: TR = 2.1 s, TE = 4.38 ms, flip angle = 8°, field of view = 220 mm, slice thickness = 1 mm, in-plane resolution = 0.86 × 0.86 mm^2^, and number of sagittal slices = 160.

Session 2 (6 hrs) and 3 (2 weeks) procedures were similar to session 1, patients filled out MPQ at all three time points. Some subjects had missing values in sensory and affective scores (n = 3 at 6 hour and n = 2 at 2 weeks) scores in the MPQ and were not included in corresponding statistical testing. This was followed by scanning procedures identical to those used at baseline.

The CBP patients in the observational group received no treatment. They filled out the McGill Pain Questionnaire at baseline and after a two-week period. They too were trained on the finger span device and had fMRI scan at their second visit (not analyzed for the present study). For this group, change in back pain was assessed between baseline and the second visit, using the visual analog scale (VAS) of the MPQ questionnaire.

### Image pre-processing and GLM analysis

Image analysis to reveal significant brain activity based on changes in blood oxygen level-dependent (BOLD) signal was performed on each patient’s data using Functional Magnetic Resonance Imaging of the Brain (FMRIB) Expert Analysis Tool [(FEAT; [41]; http://www.fmrib.ox.ac.uk/fsl)]. The data pre processing were conducted using the FSL 4.1 [41] and MATLAB 7.9. First, the skull of brain was extracted and the first 4 volumes were removed to compensate for scanner drifts. Moreover, typical FSL preprocessing was implemented which includes slice-time correction spatial smoothing with 5mm kernel, intensity normalization, and high-pass filtering (150 sec). The mean BOLD signal from white matter, cerebrospinal fluid, and whole brain without skull and the 6 motion components from motion correction, and motion outlier vectors were regarded as covariates of no interest and regressed out from the BOLD signal. In addition, probabilistic Independent Component Analysis was then implemented in MELODIC (Multivariate Exploratory Linear Decomposition into Independent Components) to select artefact components, using an automated procedure that identified and removed edge components and signal dropout components. The fMRI signal was then linearly modeled on a voxel by voxel basis using FMRIB’s Improved Linear Model (FILM) with local autocorrelation correction [42,43].

### Analysis of effects of Lidocaine vs. placebo

For this step, the experimenter was given a code that separated the subjects into two groups. However, the experimenter was not informed about the type of treatment (5% Lidocaine or placebo). The chronic back pain intensity (peak pain in spontaneous pain ratings on numerical scale and visual analog scale values) and back pain properties (sensory and affective pain qualities) were compared between the two groups at all three scan sessions using a repeated measures analysis of variance.

Brain function in the two groups was assessed for each session for ratings of spontaneous pain and for visual control ratings. Ratings were binarized relative to the mean rating of spontaneous fluctuations of back pain [39] and convolved with a canonical hemodynamic response function (gamma function: lag, 6 s; SD, 3 s). The significance of the model fit to each voxel time series was calculated, yielding statistical parametric maps for each subject and condition. All group level analyses were carried out using FEAT in a random effects analysis after the co-registration of individual scans to standard space [152 subject average Montreal Neurological Institute (MNI) space, http://www.bic.mni.mcgill.ca/cgi/icbm_view/. Average group activity map was generated for 30 subjects to ascertain the region that corresponds significantly with spontaneous pain ratings. The next averaged map was generated by subtracting the visual activity maps from the pain activity maps with a paired t-test. Subsequently, averaged maps for each group (5% Lidocaine and placebo) were generated for the three time points. Furthermore, brain activation was contrasted between the Lidocaine and placebo group at all three time points using a random effects unpaired t-test analysis. These contrasts result in *Z-*score maps of statistically significant pain-related activity across different conditions. To correct for multiple comparisons, cluster-based corrections of the *Z*-statistic images were performed. The raw *Z*-statistic images from the group analysis were thresholded at *Z*-scores ≫ 2.3. For each resulting cluster of spatially connected voxels surviving the *Z* threshold, a cluster probability threshold of *p ≪* 0.05 was applied to the computed significance of that cluster, which corrects for multiple comparisons according to Gaussian random field theory [44]. All imaging analyses were corrected for confounds due to age, sex and depression (BDI) scores.

### Interindividual differences in treatment response

To investigate inter individual differences in treatment response we selected the 2 week period as the time point of interest and calculated change in pain from baseline. The median change was used to regroup CBP into persistent and decreasing (CBPp and CBPd). The questionnaire data was analyzed to assess differences between the two groups.

## Competing interests

The author(s) declare that they have no competing interests.

## Authors’ contributions

JAH carried out statistical analysis and drafted the manuscript. MB carried out part of statistical analysis. LH participated in preprocessing fmri data, MC and EP participated in collecting data. TJS participated in study coordination and AVA conceived the study and participated in its design and coordination. All authors have read and approved the final manuscript.
